# On the discrepancy between observed and CMIP5 multi-model simulated Barents Sea winter sea ice decline

**DOI:** 10.1038/ncomms14991

**Published:** 2017-04-12

**Authors:** Dawei Li, Rong Zhang, Thomas R. Knutson

**Affiliations:** 1 The Program in Atmospheric and Oceanic Sciences, Princeton University, 201 Forrestal Road, Princeton, New Jersey 08540, USA; 2 NOAA/GFDL, 201 Forrestal Road, Princeton, New Jersey 08540, USA

## Abstract

This study aims to understand the relative roles of external forcing versus internal climate variability in causing the observed Barents Sea winter sea ice extent (SIE) decline since 1979. We identify major discrepancies in the spatial patterns of winter Northern Hemisphere sea ice concentration trends over the satellite period between observations and CMIP5 multi-model mean externally forced response. The CMIP5 externally forced decline in Barents Sea winter SIE is much weaker than that observed. Across CMIP5 ensemble members, March Barents Sea SIE trends have little correlation with global mean surface air temperature trends, but are strongly anti-correlated with trends in Atlantic heat transport across the Barents Sea Opening (BSO). Further comparison with control simulations from coupled climate models suggests that enhanced Atlantic heat transport across the BSO associated with regional internal variability may have played a leading role in the observed decline in winter Barents Sea SIE since 1979.

## Abstract (web summary)

The relative roles of external forcing versus internal variability in the Barents Sea winter sea ice decline since 1979 remain unconstrained. Here, the authors show that enhanced Atlantic heat transport into the Barents Sea associated with regional internal variability may have played a leading role.

The decline of Arctic sea ice over the satellite period has often been attributed in large part to anthropogenic climate warming. Were the observed decline to continue at its recent rate, the Arctic would likely become nearly ice-free during summer within the next decade^1^. It is well known that the simulated multi-model ensemble mean summer Northern Hemisphere (NH) sea ice extent (SIE) decline under increased anthropogenic forcing in the coupled model intercomparison project phase 5 (CMIP5) is less rapid than that observed^1^,^2^,^3^, while the CMIP5 multi-model-simulated winter NH SIE decline shows much less discrepancy with the observations^4^.

Meanwhile, substantial multi-decadal/centennial variability of Arctic sea ice cover has been inferred from high-resolution multi-century algal proxy^5^. Multi-decadal fluctuations in Arctic surface air temperature (SAT) have been found in historical records^6^,^7^, and are attributed to reduced sea ice cover due to internal variability of Atlantic inflow^8^,^9^. Low-frequency variability in winter Arctic SIE has been shown to be linked to the Atlantic multi-decadal variability in coupled climate models^10^,^11^, as well as in historical records and palaeo proxy data^12^. The rapid decline of summer Arctic SIE in recent decades may be partially due to an enhanced Atlantic heat transport into the Arctic associated with a strengthening of the Atlantic meridional overturning circulation (AMOC) since the 1970s^13^ as indicated by the observed AMOC fingerprints^14^,^15^. Both the tropical AMOC ‘fingerprint' based on the anti-correlation between surface and subsurface temperature variations in tropical North Atlantic^14^ and the extra-tropical AMOC ‘fingerprint' based on the leading mode of upper ocean heat content variations in the extra-tropical North Atlantic^15^ suggest that the AMOC has strengthened since the 1970s. The importance of internal variability is further suggested by the fact that the recent rapid decline of summer Arctic SIE took place during a period of only relatively modest global warming^3^. The Arctic SIE is also found to be subject to significant internal variability among individual ensemble members of National Center for Atmospheric Research (NCAR) CCSM3 and CCSM4 simulations forced by identical changes in anthropogenic forcing^16^,^17^. On decadal to multi-decadal time scales, internal variability could also temporarily counter changes in anthropogenic radiative forcing and cause a hiatus or pause in the decline of Arctic sea ice^13^,^18^,^19^.

The Barents Sea is located on one of the main corridors of Atlantic inflow entering the Arctic. Strong coupling between winter Barents Sea SIE and the Atlantic heat transport into the Arctic through the Barents Sea Opening (BSO) are found in both observations and modelling studies^13^,^19^,^20^,^21^,^22^. Multi-decadal variability of the Atlantic inflow temperature in the Barents Sea has also been recorded and is correlated with the Atlantic multi-decadal variability^23^,^24^. Recent work also suggests that winter Barents Sea SIE is well correlated with summer Arctic SIE in both coupled climate model simulations and observations, suggesting an important role for enhanced Atlantic heat transport into the Arctic in both winter and summer Arctic sea ice declines^13^.

In this study, we would like to address the following questions: What are the relative roles of changes in external radiative forcing versus internal variability in causing the observed winter Barents Sea SIE decline over the satellite period? Are there any substantial discrepancies between the observed and CMIP5-simulated externally forced response in winter NH sea ice decline, particularly given that the internal variability of Atlantic heat transport into the Arctic is largely removed in the CMIP5 multi-model ensemble mean forced response? The work presented here aims to provide insights on these questions by comparing observations with the CMIP5 multi-model ensemble simulations forced by changes in external radiative forcing as well as with long pre-industrial control simulations under constant radiative forcing. We show that the CMIP5 externally forced winter NH sea ice concentration (SIC) trend in individual regions (especially in Barents Sea) differs substantially from that observed. Our analysis suggests that enhanced Atlantic heat transport into the Barents Sea associated with regional internal variability may have played a leading role in the observed winter Barents Sea SIE decline since 1979.

## Results

### Discrepancies between observations and CMIP5 ensemble mean

**Figure 1:**
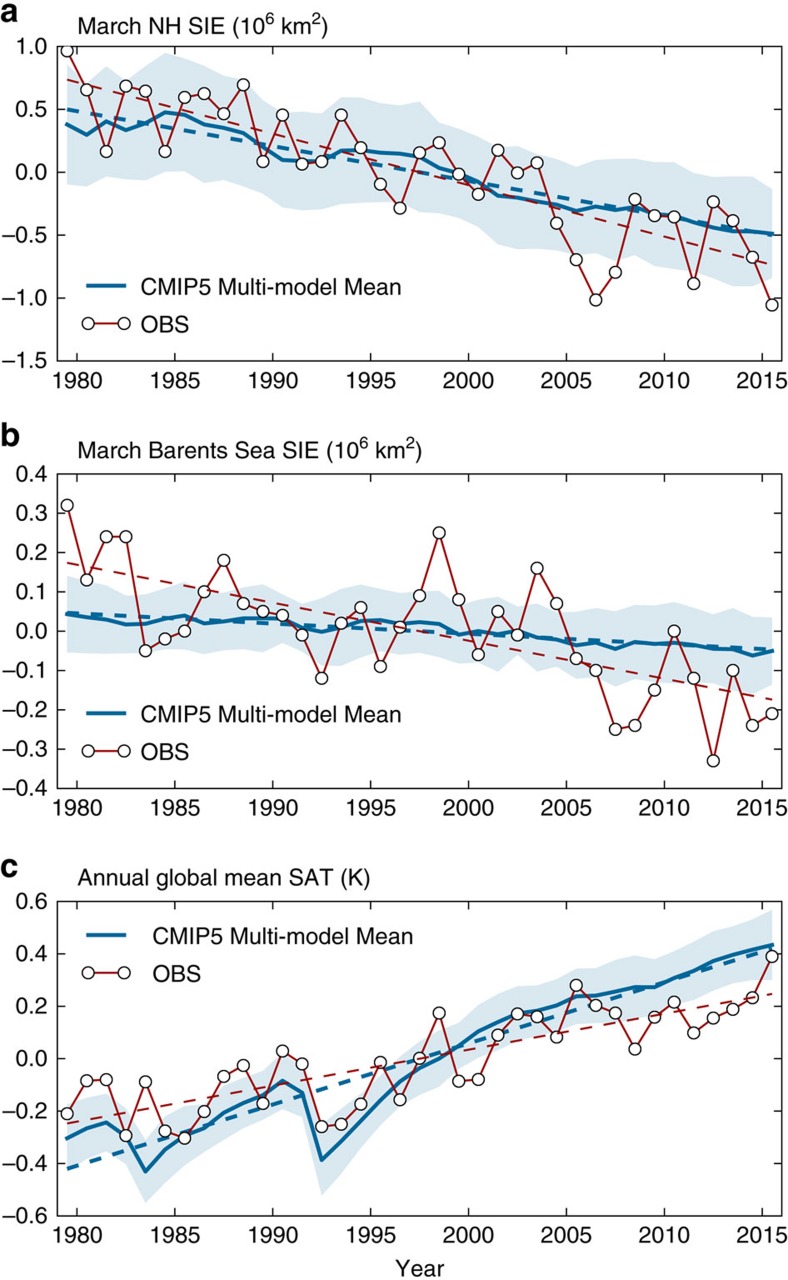
Observed and CMIP5 multi-model-simulated time series. (**a**) March NH SIE anomaly (**b**) March Barents Sea SIE anomaly (**c**) Annual global mean SAT anomaly. Blue solid line: CMIP5 multi-model ensemble mean; light blue shading: spread (±1 s.d.) across multi-model ensemble members; blue dashed line: linear trend. Observations (SIE from NSIDC and SAT from the average of three reanalysis datasets—NCEP/NCAR, ERA-Interim and MERRA) and their linear trends are shown in red solid lines with circle markers and red dashed lines, respectively.

In this study, we included 79 ensemble members from 32 CMIP5 models (‘Methods' section), and the multi-model ensemble mean represents the response to changes in external radiative forcings in the models, as the internal variability in individual ensemble members tends to cancel out in the multi-model ensemble mean. The CMIP5 multi-model ensemble mean March NH SIE shows a gradual decline over the 37-year period from 1979 to 2015 due to changes in external radiative forcing (Fig. 1a). The observed March NH SIE anomalies and associated trend generally remain within ±1 s.d. of the CMIP5 multi-model ensemble mean except the recent low records since 2005 (Fig. 1a). The CMIP5 multi-model ensemble mean March NH SIE declining trend (−1.03 million km^2^ per 37 years) is weaker than that observed (−1.51 million km^2^ per 37 years), but still accounts for a substantial part (68%) of the observed declining trend. On the other hand, the observed March Barents Sea SIE declining trend is not well captured by the CMIP5 multi-model ensemble mean. Both the observed March Barents Sea SIE declining trend and anomalies often stay outside of the ±1 s.d. of the CMIP5 multi-model ensemble mean (Fig. 1b). The CMIP5 multi-model ensemble mean declining trend of March Barents Sea SIE during this period (−0.10 million km^2^ per 37 years) is much weaker than that observed (−0.36 million km^2^ per 37 years), accounting for only a modest part (28%) of the observed declining trend.

Despite the weaker than observed declining trends in March NH and Barents Sea SIEs in the CMIP5 multi-model ensemble mean, the annual global mean SAT simulated by the CMIP5 multi-model ensemble mean displays a warming trend (0.86 K per 37 years) stronger than that observed (0.51 K per 37 years) (Fig. 1c). Both the observed global mean SAT warming trend and anomalies are often (more than half of the time) outside of the ±1 s.d. of the CMIP5 multi-model ensemble mean (Fig. 1c). Here the annual global mean SAT is a measure of the response to global scale changes in external radiative forcings, which is better represented by global mean SAT than by regional SATs. If we take into account this discrepancy in the externally forced response in global mean SAT warming trend versus observations, the externally forced response in March NH and Barents Sea SIE trends should be adjusted by the ratio 0.51/0.86≈0.59, increasing the discrepancy between the observed March NH and Barents Sea SIE trends even more.

**Figure 2:**
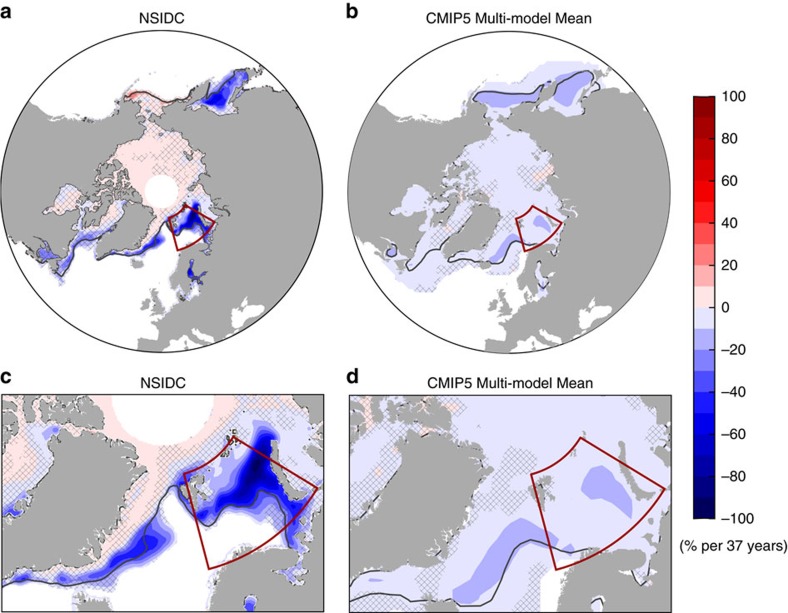
Spatial pattern of observed and CMIP5 multi-model-simulated March SIC trend over 1979–2015. (**a**,**c**) Observations (NSIDC), (**b**,**d**) CMIP5 multi-model ensemble mean. The lower panels zoom in over the Barents Sea. Black lines mark the climatological March sea ice edge, and the red box outlines the Barents Sea. SIC from individual CMIP5 models are regridded onto a common polar-stereographic grid so that a multi-model ensemble mean is applicable. Grid points with a mean SIC less than 1% are masked white. In **a**,**c**, grid points poleward of 85°N are also masked due to missing data. The unhatched regions in all panels have statistically significant trends at 95% confidence level.

Why does the CMIP5 externally forced response account for a substantial part of the observed March NH SIE trend, but only a modest part of the observed March Barents Sea SIE trend (Fig. 1)? We interpret this in terms of the discrepancy between observed and CMIP5 multi-model-simulated spatial patterns of March NH SIC trends over the satellite period (1979–2015). The observed declining trend in March NH SIC is most pronounced in the northeast section of the Barents Sea (Fig. 2a,c). Beyond the Barents Sea, the observed March NH SIC trend also exhibits significant decline in the Sea of Okhotsk and Greenland Sea, but shows a slight increase in the Bering Sea and central Arctic (Fig. 2a). The observed increase of SIC in the Bering Sea was also found in a previous study^25^, but is not statistically significant in this analysis with up-to-date data. In contrast, the CMIP5 externally forced response in March NH SIC displays a much weaker declining trend in regions where the observed decline is most pronounced (Fig. 2b,d). It also exhibits a weak declining trend over a broader region in the NH, and thus an opposite trend to that observed in central Arctic (Fig. 2a,b). On the other hand, although the CMIP5 externally forced March NH SIC trends in individual regions differ substantially from those observed (Fig. 2), the CMIP5 externally forced declining trend in March NH SIE is not much weaker than that observed (Fig. 1a) because the discrepancy in the spatial pattern is masked by summing over the entire NH. Hence, simply comparing the time series of March NH SIE anomalies is insufficient for understanding the cause of the observed winter NH sea ice decline. Closer inspection of spatial patterns of NH SIC trends reveals why the CMIP5 externally forced response compares much better with the observed March NH SIE declining trend than with the observed March Barents Sea SIE declining trend (Fig. 1a,b). A better performance in simulating the observed time series of winter NH SIE can be misleading, as the model-simulated spatial structures may differ substantially from observations. Similar results are also found in a previous study showing forced ensemble mean sea ice response from one CMIP5 model^19^.

### Impact of the Atlantic heat transport in CMIP5 simulations

**Figure 3:**
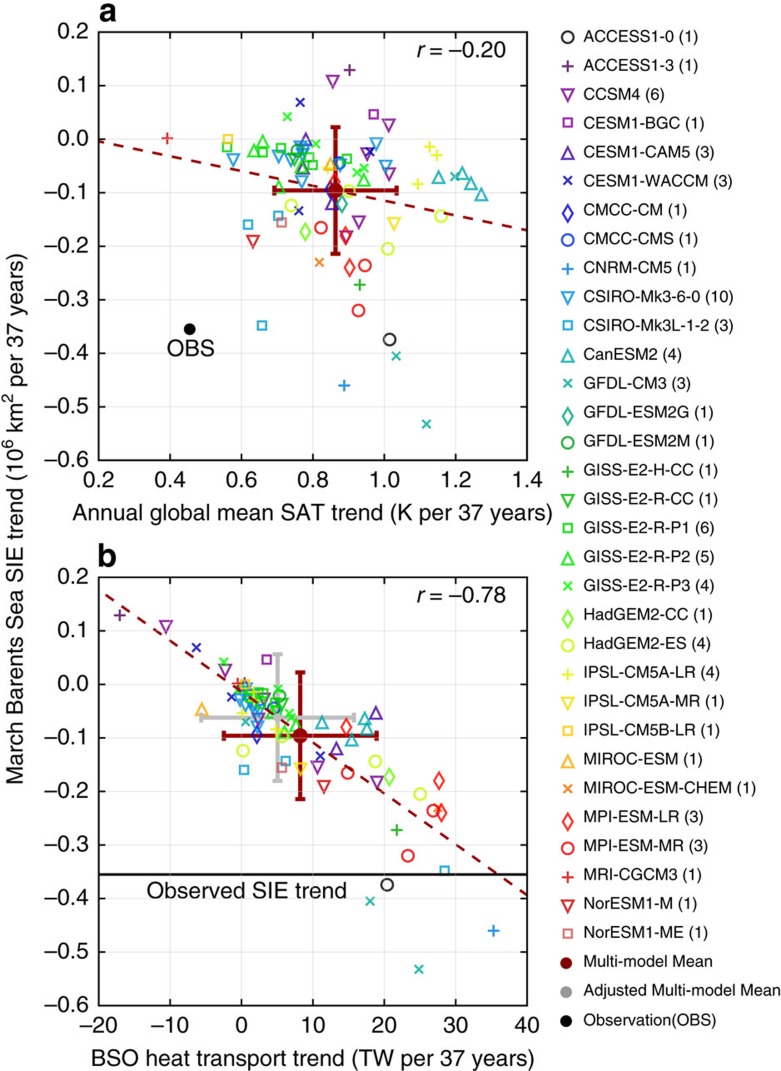
Scatter plots of trends over 1979–2015 from CMIP5 multi-model ensemble members. (**a**) March Barents Sea SIE trend versus annual global mean SAT trend (**b**) March Barents Sea SIE trend versus trend of annual mean Atlantic heat transport across the BSO (HT_BSO_). In parentheses (model list) is the number of ensemble members for each model. Solid red circles: CMIP5 multi-model ensemble mean trends. Thick red crosses: spread (±1 s.d.) across multi-model ensemble members. Grey solid circle and thick grey cross in **b**: adjusted multi-model mean trends and spread (see text). The solid black circle in **a** denotes the observed trends. The solid black line in **b** marks the observed March Barents Sea SIE trend. Red dashed lines: least-square linear fits.

There is no significant anti-correlation (*r*=−0.2) between annual global mean SAT trends and March Barents Sea SIE trends over the 37-year period across the CMIP5 ensemble members (Fig. 3a). In contrast, March Barents Sea SIE trends are strongly anti-correlated with the trends in the annual mean Atlantic heat transport across the BSO (HT_BSO_) (*r*=−0.78, Fig. 3b) over the 37-year period across the CMIP5 ensemble members. The results suggest that an enhanced HT_BSO_ has played a key role in the March Barents Sea SIE decline while the warming in global mean SAT appears not important for March Barents Sea SIE decline in individual CMIP5 ensemble members. Furthermore, there is strong positive correlation (*r*=0.69) between the trends in annual mean net upward surface heat flux integrated over the Barents Sea region (F_SFC_) and trends in HT_BSO_, and strong anti-correlation (*r*=−0.64) between trends in F_SFC_ and trends in March Barents Sea SIE, over the 37-year period across the CMIP5 ensemble members. Hence, an enhanced HT_BSO_ leads to a reduced winter sea ice cover in the Barents Sea, and more net upward surface heat flux is released into the atmosphere through larger winter open water area in the Barents Sea^20^,^26^,^27^. Through this mechanism, changes in F_SFC_ provide a negative feedback to changes in winter Barents Sea SIE. The positive correlation between HT_BSO_ and F_SFC_ trends and the negative correlation between F_SFC_ and March Barents Sea SIE trends suggests that at multi-decadal time scales, changes in HT_BSO_ are forcing changes in Barents Sea SIE and F_SFC_ in the models, not the other way around. If the net upward surface heat flux (F_SFC_) were driving the winter Barents Sea SIE changes, one would expect a positive correlation between them (that is, sea ice loss occurs when net downward surface heat fluxes are enhanced), which is opposite to the behaviour simulated in the models.

**Figure 4:**
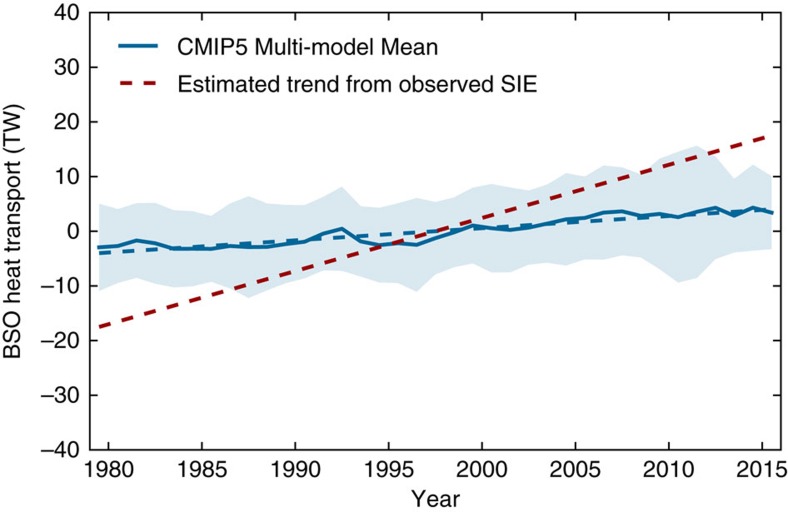
Time series of CMIP5 multi-model-simulated annual mean HT_BSO_ anomaly. Blue solid line: CMIP5 multi-model ensemble mean; light blue shading: spread (±1 s.d.) across multi-model ensemble members; blue dashed line: linear trend. Red dashed line: estimated annual mean HT_BSO_ trend (∼35 TW per 37 years) implied from the observed SIE trend in Fig. 3b.

The CMIP5 multi-model ensemble mean forced response in HT_BSO_ does show a positive trend (∼8 TW per 37 years, Fig. 4). If we take into account the overestimation of the externally forced response in CMIP5 multi-model ensemble mean (Fig. 1c), the externally forced response in HT_BSO_ should also be adjusted by the ratio 0.51/0.86=0.59, and the inferred externally forced increase of HT_BSO_ over the 37-year period should be 8 TW × 0.59≈5 TW after the adjustment. In comparison, the HT_BSO_ increase needed to induce the observed March Barents Sea SIE declining trend over the 37-year period is on the order of 35 TW, as indicated by the least-square linear-fit line of the scatter plot (Fig. 3b). This is consistent with the simulated HT_BSO_ increasing trend of ∼35 TW over this period in the decadal prediction experiments initialized with the observed ocean information and the ocean-only simulation forced by observed atmospheric states in a recent study^19^. The adjusted CMIP5 externally forced HT_BSO_ increase (∼5 TW per 37 years) is thus too small to cause the observed decline in winter Barents Sea SIE (Fig. 4). Based on CMIP5 results and the observed March Barents Sea SIE declining trend (Fig. 3b), we estimate that an increase in HT_BSO_ on the order of 30 TW per 37 years (that is, 35 TW per 37 years–5 TW per 37 years) due to regional internal variability is necessary to explain the dramatic decrease of winter Barents Sea SIE seen in recent decades, in addition to the externally forced response. Here internal HT_BSO_ changes could be affected both by local variability in surface winds through changes in volume transport across the BSO, as well as by remote variability in the large scale ocean circulation, such as AMOC, through changes in both volume transport across the BSO and ocean temperature of the Atlantic inflow.

### Impact of the Atlantic heat transport in control simulations

**Figure 5:**
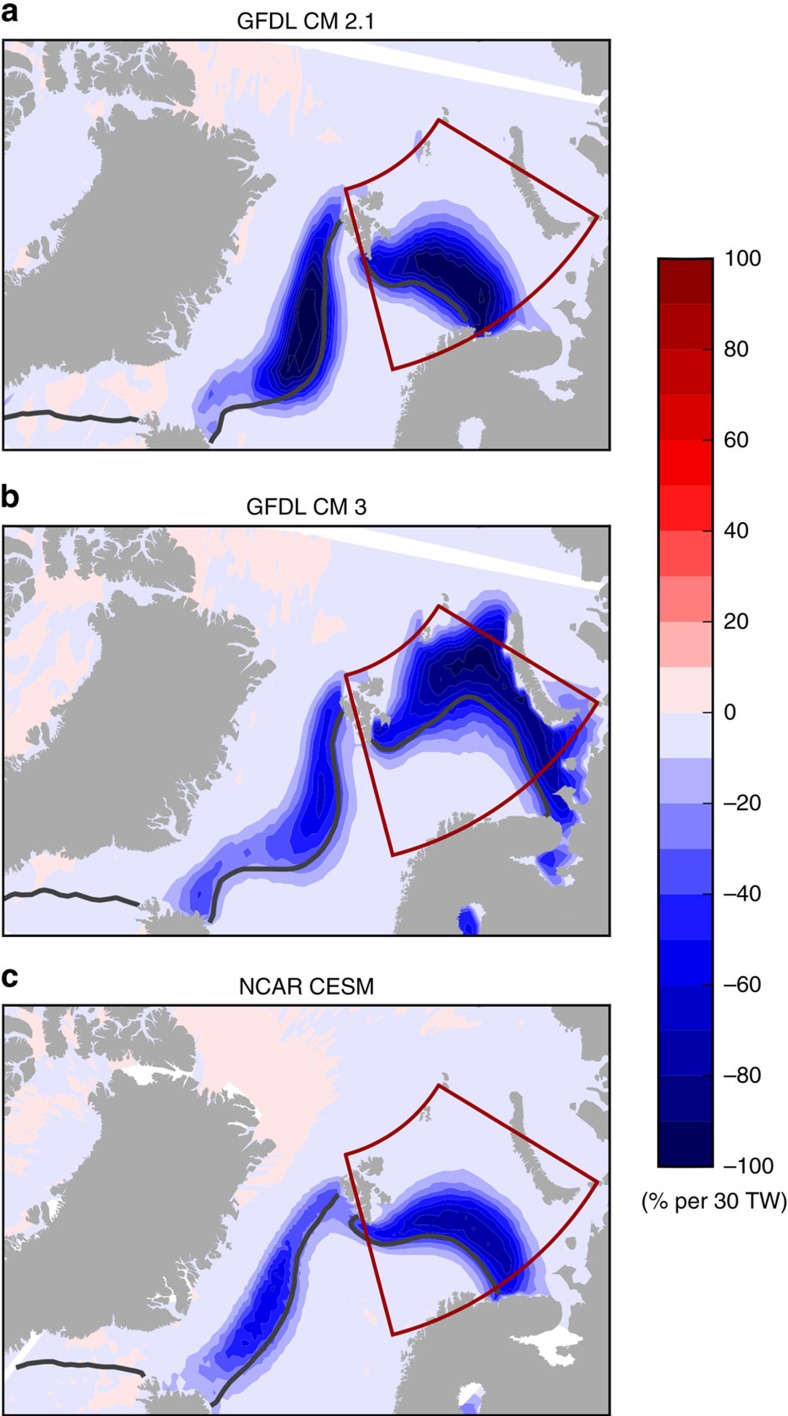
Regression of 37-year March SIC trends on 37-year HT_BSO_ trends in three long control simulations. (**a**) GFDL CM2.1 (**b**) GFDL CM3 (**c**) NCAR CESM. Red boxes outline the Barents Sea region, and black lines denote the climatological March sea ice edge in each model. The regression maps are scaled to correspond to a positive trend of 30 TW per 37 years in HT_BSO_. White gaps in the upper right corners of **a**,**b** are due to the polar projection of SIC simulated on tri-polar grids in GFDL models.

To further investigate the response of winter Barents Sea SIE to internal variability in Atlantic heat transport across the BSO (HT_BSO_), we seek insights from long-term pre-industrial control simulations that are available from three coupled climate models (GFDL CM2.1, GFDL CM3 and NCAR CESM). As discussed in the previous section, we estimate that an increase in HT_BSO_ on the order of 30 TW per 37 years due to regional internal variability is necessary to explain the dramatic decrease of winter Barents Sea SIE seen in recent decades. The simulated March Barents Sea SIC declines in response to a positive trend of HT_BSO_ (scaled to 30 TW per 37 years) in all three models due to internal variability (Fig. 5) are much larger than the CMIP5 multi-model ensemble mean forced response, and are on the same order as that observed (Fig. 2c,d). GFDL CM3 exhibits a spatial pattern closest to that observed with the most pronounced decline of March SIC in the northeast section of the Barents Sea, partially because its climatological March SIC and sea ice edge is more similar to that observed during recent decades (Figs 2c and 5). The control simulations from GFDL CM2.1 and NCAR CESM both have excessive climatological March sea ice in the Barents Sea region, thus the most responsive area of March SIC is displaced towards the southwest section of the Barents Sea (Fig. 5). The correlations between 37-year March Barents Sea SIE trends and HT_BSO_ trends of 500 randomly sampled 37-year segments from each control simulation are −0.94, −0.96 and −0.91 for GFDL CM2.1, GFDL CM3 and NCAR CESM, respectively, larger than the CMIP5 inter-model correlation (−0.82, Fig. 3b). The regression coefficients between 37-year March Barents Sea SIE trends and HT_BSO_ trends are −0.31, −0.55 and −0.25 million km^2^ per 30 TW for GFDL CM2.1, GFDL CM3 and NCAR CESM, respectively. The response of March Barents Sea SIE trends to HT_BSO_ trends in GFDL CM2.1 is about the same as that derived from the CMIP5 ensemble members (−0.31 million km^2^ per 30 TW, Fig. 3b), while the response is stronger in GFDL CM3 and weaker in NCAR CESM compared to the CMIP5 ensemble.

The CMIP5 individual ensemble members in general seem incapable of simulating a positive HT_BSO_ trend on the order of 30 TW per 37 years. The majority of CMIP5 ensemble members have much smaller trends in both HT_BSO_ and March Barents Sea SIE (Fig. 3b). Among the three long control simulations, the largest trends of HT_BSO_ over all available 37-year segments are 23, 45, and 25 TW per 37 years in the three control simulations (GFDL CM2.1, GFDL CM3 and NCAR CESM), respectively. Even for GFDL CM3, only 38 out of all 3,564 available 37-year segments have such large HT_BSO_ trends (⩾30 TW per 37 years), suggesting a rare occurrence. The implications are that either current climate models have unrealistically small amplitudes of low-frequency internal variability in HT_BSO_, or the recent observed decline of March Barents Sea SIE is a remarkably rare event. We speculate that it is more likely that models have unrealistically small amplitudes of low-frequency internal variability in HT_BSO_ due to common systematic model deficiencies and mean state biases, rather than that the recent observed decline is a rare event. The underestimation of mean state HT_BSO_ is implied from the simulated excessive mean state winter Barents Sea SIE (southward shift of climatological March sea ice edge) in CMIP5 models (Fig. 2b,d), as also found in CMIP3 models in a previous study^28^. Under the same atmospheric forcing, a high-resolution regional ocean model simulates stronger mean state HT_BSO_ than a low-resolution global model does^29^.

## Discussion

In this study, we show that a comparison of the observed and CMIP5 multi-model-simulated time series of March NH SIE anomalies alone is insufficient for understanding the cause of the observed winter NH sea ice declining trend over the satellite period. Closer inspection of spatial patterns of NH SIC trends reveals that the CMIP5 externally forced March NH SIC trend in individual regions differs substantially from that observed. The CMIP5 externally forced response in March NH SIC displays a much weaker decline in regions where the observed decline is most pronounced (Barents Sea). It also exhibits a weak declining trend over a broader region in the NH, and thus opposite to the observed increasing trend in central Arctic. Hence, the CMIP5 externally forced response in March Barents Sea SIE exhibits a large discrepancy (that is, a much smaller decline) compared to that observed, but the discrepancy is less severe in March NH SIE because the discrepancies in the spatial pattern are partially masked by summing over the entire NH. The large discrepancies in the spatial pattern of March NH SIC trends and in March Barents Sea SIE trend suggest that the observed winter NH sea ice trend over the satellite period cannot be well explained by the externally forced response alone, and that the observed rapid declining trend in winter Barents Sea SIE is not predominantly due to external radiative forcing.

Our analysis further suggests that an enhanced Atlantic heat transport across the BSO (HT_BSO_) has played a key role in March Barents Sea SIE decline, while the warming in global mean SAT has been relatively unimportant for March Barents Sea SIE decline in individual CMIP5 ensemble members. We identify a significant high anti-correlation between winter Barents Sea SIE trends and HT_BSO_ trends throughout individual CMIP5 ensemble members. This suggests that at multi-decadal time scales, changes in HT_BSO_ are forcing changes in Barents Sea SIE and in net upward surface heat flux over the Barents Sea in these models. The CMIP5 externally forced HT_BSO_ increase is too small to cause the observed decline in winter Barents Sea SIE. Based on CMIP5 results and observations, we estimate that a total 35 TW per 37 years trend in HT_BSO_ is needed to explain the observed winter Barents Sea SIE decline over the satellite period. Among this, an increase in HT_BSO_ on the order of 30 TW per 37 years due to regional internal variability is necessary to explain the majority of the observed winter Barents Sea SIE decline seen in recent decades and its discrepancies with the CMIP5 multi-model mean forced response. Results from long pre-industrial control simulations of three coupled models show that the simulated March Barents Sea SIC declines in response to a positive trend of HT_BSO_ (scaled to 30 TW per 37 years) are much larger than the CMIP5 multi-model ensemble mean forced response and are on the same order as that observed.

To our knowledge, our work is the first to use CMIP5 coupled climate models, augmented by extended control simulations, to quantify the relative contribution of external radiative forcing versus internal variability to the observed Barents Sea winter SIE decline. Our results suggest that enhanced HT_BSO_ associated with regional internal variability may have played a leading role in the observed decline in winter Barents Sea SIE over the satellite period. Further studies are needed to provide additional support for this proposed mechanism. Understanding the leading cause of the observed winter Barents Sea SIE decline over the satellite period is crucial for understanding and predicting the associated broad impacts on high/mid latitude climate and weather, and Arctic ecosystems.

## Methods

### Observational data

Here we use the satellite SIC data for the 37-year period (1979–2015) from the National Snow and Ice Data Center (NSIDC)^30^. We define the NH and Barents Sea SIEs as the total marine area where SIC ≥15% within the NH and within the Barents Sea (70° N–81° N, 15° E–60° E), respectively. The climatological sea ice edge is defined as the 15% climatological SIC contour line.

The observed global mean SAT is derived from the averaged SAT of three reanalysis data sets: NCEP/NCAR^31^, ERA-Interim^32^ and NASA MERRA^33^. The annual global mean SAT trends over the 37-year period in three reanalysis data sets are: NCEP/NCAR (0.61 K per 37 years), ERA-Interim (0.46 K per 37 years) and NASA MERRA (0.45 K per 37 years), and the averaged trend of three reanalysis data sets is (0.51 K per 37 years). It has been shown that SAT data in NASA MERRA is most realistic in the Arctic region^34^.

### CMIP5 all-forcing simulations

To compare with the observations, we combined CMIP5 twentieth century all-forcing (historical) simulations with CMIP5 representative concentration pathway (RCP) 4.5 future emission scenario simulations to cover the period from 1979 to 2015. The RCP4.5 pathway is chosen from among the four available future emission scenarios to represent the period after 2005, noting that these scenarios deviate little from each other during the short period 2006–2015. A total of 79 ensemble members from 32 CMIP5 models are selected, based on the criteria that all variables needed in our analyses are available in a selected ensemble member, and that the ensemble member has both historical and RCP4.5 simulations so that the they can be combined. Due to the large number of selected ensemble members, the multi-model ensemble means represent the response to changes in external radiative forcings in the models, as the internal variability in individual ensemble members tends to cancel out in the multi-model ensemble means. In this study, both observed and CMIP5-simulated anomalies are referenced to climatological means for 1979–2015. The correlation between March and November–April averaged Barents Sea SIE trends over the 37-year period across CMIP5 ensemble members is 0.94, and the correlation between March and February–April averaged Barents Sea SIE trends over the 37-year period across CMIP5 ensemble members is 0.99. These confirm that trends in March SIE are representative for trends in the winter SIE in this study. To study the oceanic impact on the Barents Sea winter SIE, we calculated the annual mean advective Atlantic heat transport across the BSO (71° N–76° N, 20° E) (HT_BSO_) using monthly mean ocean velocities and potential temperature (reference temperature being 0 °C) from the selected CMIP5 ensemble members.

### Long-term control simulations

Additionally, long-term control simulations available from three coupled climate models under constant pre-industrial radiative forcing are employed to assess the impact of internal variability in HT_BSO_ on Barents Sea winter SIE. The models used here are the Geophysical Fluid Dynamics Laboratory (GFDL) Coupled Model version 2.1 (CM2.1)^35^, GFDL Coupled Model version 3 (CM3)^36^,^37^ and the NCAR Community Earth System Model (CESM)^38^. Only 1,801 years of output are available from the NCAR CESM control simulation, while much longer control simulations (3,600-year segments) are available from GFDL CM2.1 and GFDL CM3. To derive the changes in SIC associated with the internal variability in HT_BSO_, 500 random samples of 37-year segments (same length as 1979–2015) are drawn from the control simulations, and the 500 pairs of 37-year trends in SIC and HT_BSO_ are obtained for each model, respectively. Then SIC trends are regressed onto HT_BSO_ trends for each grid point to obtain the spatial pattern of SIC changes associated with HT_BSO_ changes due to simulated internal variability in each model.

### Data availability

All CMIP5 model output are available from https://pcmdi.llnl.gov/search/cmip5. NSIDC sea ice satellite sea ice can be downloaded from http://nsidc.org/data/NSIDC-0051. NCEP/NCAR reanalysis data can be downloaded from http://www.esrl.noaa.gov/psd/data/gridded/data.ncep.reanalysis.html. Other data and code are available from the authors on request.

## Additional information

**How to cite this article:** Li, D. *et al.* On the discrepancy between observed and CMIP5 multi-model simulated Barents Sea winter sea ice decline. *Nat. Commun.* **8,** 14991 doi: 10.1038/ncomms14991 (2017).

**Publisher's note**: Springer Nature remains neutral with regard to jurisdictional claims in published maps and institutional affiliations.
